# Clinical and imaging features of tufted angioma in children

**DOI:** 10.3389/fped.2026.1756736

**Published:** 2026-06-18

**Authors:** Bingxuan Jiao, Dan Song, Liang Wang

**Affiliations:** Department of Vascular Anomalies and Interventional Radiology, Children’s Hospital Affiliated to Shandong University (Jinan Children’s Hospital), Jinan, China

**Keywords:** clinical findings, computed tomography, magnetic resonance imaging, tufted angioma, ultrasound

## Abstract

**Introduction:**

Tufted angioma (TA) is a rare vascular tumor, and its imaging characteristics have rarely been reported. This study aimed to investigate the clinical and imaging features of TA to provide a reference for its early diagnosis.

**Methods:**

Between March 2022 and August 2024, the clinical and imaging findings of TA, initially diagnosed based on clinical manifestations and confirmed by histopathology, were retrospectively analyzed.

**Results:**

The mean age of the five children was 2.2 years (range 0.6–9.4 years), including two boys and three girls. The symptoms mainly consisted of cutaneous lesions associated with erythematous skin changes. Ultrasonography showed superficial hypoechoic masses with increased echogenicity of the underlying soft tissue. On computed tomography, the lesions appeared as skin thickening and flocculent soft-tissue shadows, demonstrating striated and/or flocculent significant homogeneous enhancement on contrast-enhanced scans. All TAs showed homogeneous hyperintensity on fat-saturated T2-weighted images without significant diffusion restriction on diffusion-weighted imaging.

**Discussion:**

TA primarily occurs in infants and can arise at various body locations. Its imaging manifestations include ill-defined superficial lesions with subcutaneous adipose tissue involvement and significant enhancement, which may serve as a reference for clinical diagnosis.

## Introduction

Tufted angioma (TA) is a rare vascular tumor that typically occurs during childhood. It typically presents as a solitary lesion, although cases with multiple lesions have also been reported (1, 2). TA often affects the neck, trunk, and extremities (3). Some cases are associated with the Kasabach–Merritt phenomenon (KMP), an aggressive and life-threatening condition characterized by rapid tumor growth, severe platelet consumption, and consumptive coagulopathy (4, 5). Previous studies have reported a mortality rate of up to 30%–37.5% in patients with KMP because of bleeding complications, high-output cardiac failure, and infiltration of vital organs (6, 7). Therefore, early diagnosis and timely intervention may help retard TA progression and save the lives of patients. To date, studies of TA have mainly been case reports, with limited findings on its ultrasonographic, computed tomography (CT), and magnetic resonance imaging (MRI) features. Thus, this retrospective study aimed to review the clinical and imaging features of TA to enhance the understanding of the disease by physicians and radiologists and to provide a reference for its early diagnosis.

## Methods

### General information

This study was approved by the Ethics Committee of the Children's Hospital Affiliated to Shandong University. Written informed consent was obtained from the guardians of all children. Children diagnosed with TA and admitted to the Children's Hospital Affiliated to Shandong University between March 2022 and August 2024 were retrospectively enrolled. Clinical characteristics, including sex, age at onset, medical history, symptoms, anatomical location, lesion extent, and laboratory findings, were collected. The diagnosis of TA was initially based on clinical manifestations and imaging findings and was then confirmed by histopathology. Lesion locations were categorized as the head and neck, trunk, and extremities.

### Imaging information

All five children with TA underwent ultrasonographic examination. In addition, four underwent contrast-enhanced CT and one underwent enhanced MRI scan. Two radiologists, each with more than 8 years of experience in pediatric radiology, independently evaluated the following characteristics: lesion location, size, shape, extent, internal structure, homogeneity, and borders. Based on lesion extent, the lesions were classified into two subtypes: the focal type, defined as a solitary lesion with surrounding infiltration, and the infiltrative type, described as a diffuse lesion with ill-defined borders of the solid component.

The echo intensity, CT density, and MRI signal intensity of TA were classified as low, moderate, and high, respectively, by comparison with those of muscle. For contrast-enhanced CT and MRI, enhancement approximately equivalent to that of muscle was defined as mild enhancement; enhancement comparable to that of the blood vessels was defined as significant enhancement; and enhancement between the mild and significant enhancement was classified as moderate enhancement. The protocols for CT and MRI scans are provided in Supplementary Material 1. All images were assessed by two radiologists, and the findings were recorded after consensus was reached.

## Results

### Patient characteristics

Among the five children with TA, the mean age was 2.2 years (range 0.6–9.4 years). Two patients were boys, with ages at onset ranging from 6 to 8 months, and three were girls, with ages at onset ranging from 1 week to 6 months. The symptoms mainly included lumps on the surface of the skin (5/5), erythematous skin changes (5/5), and tenderness (1/5).

All five children were born at full term. Regarding lesion location (Table 1), two lesions were located on the trunk, one on an extremity, and one on the lower jaw, with one case exhibiting involvement affecting the head and neck as well as the chest and posterior chest wall of the trunk simultaneously. Blood test (Table 1) showed decreased fibrinogen levels with normal D-dimer levels in three children, while one had an increased D-dimer level with a normal fibrinogen level. One child had normal fibrinogen and D-dimer levels. None of the children exhibited thrombocytopenia.

**Table 1:** Clinical features of all patients.

Patients	Age	Gender	Lesion location	Fibrinogen	D-dimer	Thrombocytopenia
1	1 years 2 months	Female	Right shoulder	D	N	No
2	6 months	Female	Chin	D	N	No
3	7 months	Female	Chest wall	D	N	No
4	15 years	Male	Head, neck, and chest wall	N	I	No
5	3 years 10 months	Male	Chest wall	N	N	No

N, normal; I, increase; D, decrease.

### Ultrasound features

All five children presented with a hypoechoic mass within the skin layer with increased echogenicity of the underlying subcutaneous fat layer (Table 2). Of all tumor-like lesions, all five cases had ill-defined borders; four showed abundant blood flow signals, one showed patchy blood flow signals, three presented anechoic tubular lesions within the areas of increased echogenicity, and all five showed skin thickening. The mean depth of the overall region involved was 0.9 ± 0.2 cm.

**Table 2:** Imaging features of all patients.

Patients	US			CT			
	BLS	ATL	ST	LF	ST	FSS	PSISAT
1	Abundant	Yes	Yes	Diffuse	Yes	Yes	Yes
2	Abundant	Yes	Yes	Diffuse	Yes	Yes	Yes
3	Abundant	No	Yes	Focal	Yes	Yes	No
4	Patchy	No	Yes	Diffuse	Yes	Yes	Yes
5	Abundant	Yes	Yes	-	-	-	-

US, ultrasound; CT, computed tomography; BLS, blood flow signal; ATL, anechoic tubular lesion; ST, skin thickening; LF, lesion feature; FSS, flocculent soft-tissue shadow; PSISAT, punctate soft-tissue intensity within subcutaneous adipose tissue.

### CT findings

**Figure 1:**
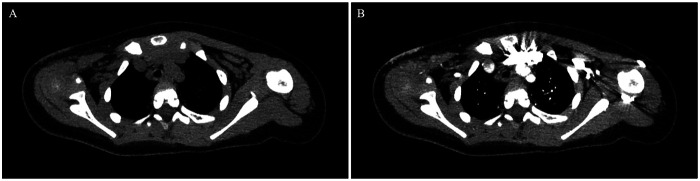
A girl with tufted angioma, aged 1 year and 2 months. **(A)** Plain CT showing cutaneous flocculent soft-tissue shadows and punctate soft-tissue intensity within subcutaneous adipose tissue. **(B)** Contrast-enhanced CT showing heterogeneous enhancement. CT, computed tomography.

Among the four children who underwent CT scans (Table 2), one showed a focal lesion and three showed diffuse lesions. The involved tissues showed skin thickening (4/4), flocculent soft-tissue shadows (4/4), and punctate soft-tissue densities within the subcutaneous adipose tissue (3/4) (Figure 1A). None of the children showed involvement of the spatium intermusculare or bone invasion. The mean CT value was 29.58 ± 11.53 Hounsfield units (HU). After intravenous injection of contrast agent, the lesions showed striated and/or flocculent significant heterogeneous enhancement (4/4) (Figure 1B) in the arterial phase, with a CT value of 153.91 ± 52.11 HU.

### MRI findings

**Figure 2:**
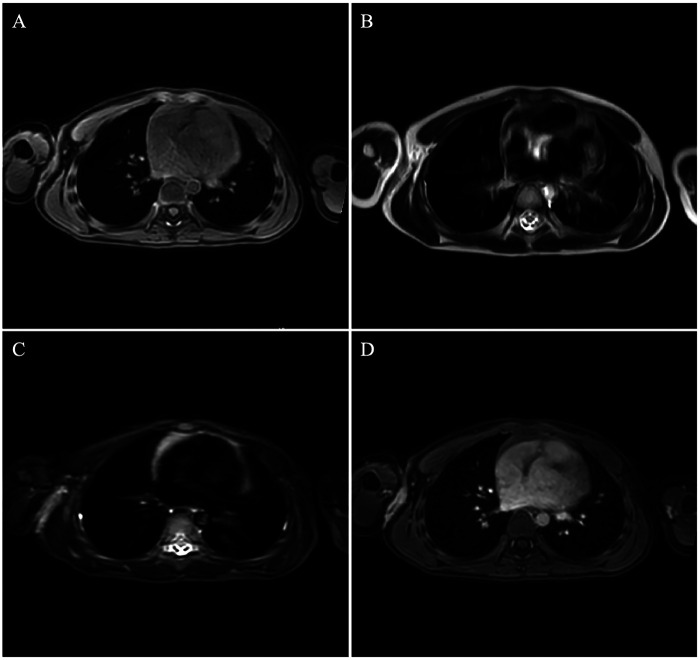
A boy with tufted angioma, aged 3 years and 10 months. TA lesion showed high, heterogeneous signal intensity on both T1WI **(A)** and T2WI **(B)** compared with muscle, while it demonstrated homogeneous, high signal intensity on fat-saturated T2WI **(C)**. **(D)** Contrast-enhanced scan showing significant homogeneous enhancement. TA, tufted angioma; T1WI, T1-weighted imaging; T2WI, T2-weighted imaging.

Only 1 child underwent an MRI scan (Figure 2). TA showed high, heterogeneous signal intensity on both T1-weighted imaging (T1WI) and T2-weighted imaging (T2WI) compared with muscle, while it demonstrated homogeneous, high signal intensity on fat-saturated T2WI. No significant diffusion restriction was observed on diffusion-weighted imaging. On contrast-enhanced fat-suppressed T1WI, TA showed significant homogeneous enhancement compared with that of blood vessels. No flow void signals, bone changes, calcification, hemorrhage, or hemosiderosis were observed.

## Discussion

TA, also known as angioblastoma of Nakagawa, was first reported by Nakagawa in 1949 (8, 9). It is a rare benign vascular tumor that most commonly occurs in infants and children (10). In this study, the age at onset ranged from 1 week to 8 months. The pathogenesis of TA remains unknown, although some reports suggest that acquired TA is related to vascular proliferative conditions, including pregnancy (11, 12). Previous studies (3, 13) have shown that the neck, trunk, and extremities are the most common sites of TA, which is consistent with our study. To some extent, TA is difficult to differentiate from kaposiform hemangioendothelioma (KHE) because of their overlapping clinical and histopathologic features and their shared association with the development of KMP (14). Therefore, hematological tests and imaging examinations are important for infants and children presenting with suspicious symptoms.

TA is often associated with skin thickening, manifesting as erythematous skin changes and indurated plaques with ill-defined margins (4, 10). The evolution of TA may involve spontaneous regression, stability, or progressive enlargement, which differs from that of KHE (15). In addition, TA is less frequently associated with KMP than KHE.TA may sometimes present with tenderness, which is more common in patients who develop KMP (16).

KMP, first reported by Kasabach and Merritt in 1940, was described as an enlarging vascular tumor complicated by severe thrombocytopenia and consumptive coagulopathy (17). Subsequent studies have shown that TA can be associated with KMP (14, 18). The incidence may be as high as 38% (12), although none of the children in this study developed KMP. The pathogenesis of KMP remains poorly understood. Thrombocytopenia does not result from impaired platelet production in the hematopoietic system. Lei et al. (4) reported that bone marrow examination of TA patients showed normal hematopoiesis, with no signs of altered erythropoiesis or megakaryocytopoiesis.

Color Doppler ultrasonography is the first choice for the evaluation of superficial masses. TA mostly appears as a heterogeneous lesion with primarily mixed echogenicity, characterized by hypoechogenicity in the superficially subcutaneous and hyperechogenicity in the underlying subcutaneous fat layer (19). In this study, hypoechogenicity and hyperechogenicity were observed in all five (100%) children. Furthermore, we observed that TA generally had no clear boundaries on ultrasound and most lesions are localized skin thickening with superficial hypoechoic lesions and abundant blood flow signals. Another study by Xiao et al. (16) showed that TA presented as multiple thickened cutaneous layers with increased blood supply on Doppler ultrasonography, which is consistent with this study. In our study, all children showed skin thickening, and four (80%) demonstrated abundant blood flow signals. For all five children, the depth of all lesions did not exceed 1 cm, which may help differentiate TA from KHE.

In this study, TA presented as flocculent soft-tissue shadows with ill-defined borders on plain CT images, and most children (75%) showed involvement of the subcutaneous adipose tissue involved. After contrast administration, TA manifested as significant heterogeneous enhancement in the arterial phase, with the degree of enhancement approximately equal to that of the surrounding blood vessels. Little literature has mentioned the CT features of TA. Lei et al. (4) described TA as a low-density mass with vague margins on CT. Unfortunately, no information regarding contrast-enhanced CT findings was provided. Mehrotra et al. (20) reported a 1.5-year-old girl who was initially suspected of having infantile hemangioma (IH) but was finally diagnosed with TA. According to Weber et al. (21), IH has well-defined borders without involvement of the surrounding adipose tissue, which may help differentiate it from TA.

The reported MRI features of TA typically include poorly demarcated lesions with predominantly homogeneous, high signal intensity on T2WI (10, 16, 19). MRI is also helpful in differentiating TA from IH. On MRI, IH presents as a well-defined lesion with homogeneous, hyperintense signal intensity on T2WI (21). On contrast-enhanced T1WI, TA showed significant homogeneous enhancement compared with blood vessels. Both CT and MRI are reliable modalities for detecting vascular lesions. CT has the additional benefit of being less time consuming, while also allowing comprehensive evaluation of blood vessels and identification of surrounding fat abnormalities, which may aid in the initial diagnosis and subsequent treatment of TA.

This study has several limitations. First, the sample size was relatively small because of the rarity of TA. As an exploratory study, the findings presented here should be viewed as preliminary evidence and require validation in larger cohorts. Second, the findings of this study are based solely on Asian children, which may differ from those in other ethnic groups.

## Conclusion

In conclusion, this study showed that TA predominantly occurred in infants and could arise at various body locations. The imaging findings demonstrated significantly ill-defined superficial lesions with subcutaneous adipose tissue involvement and significant enhancement. The results of this study may be helpful in the clinical diagnosis of TA.

## Data Availability

The raw data supporting the conclusions of this article will be made available by the authors, without undue reservation.
