# Supplementary material for: Clinical and imaging features of tufted angioma in children
Source: Front Pediatr. 2026 Jun 18;14:1756736. doi: 10.3389/fped.2026.1756736 (PMC13323123; doi:10.3389/fped.2026.1756736)
Supplement: Supplementary file 1 [file Table1.docx]

Table 1. Clinical features of all patients

| Patients | Age | Gender | Lesion Location | Fibrinogen | D-dimer | thrombocytopenia |
| --- | --- | --- | --- | --- | --- | --- |
| 1 | 1 years  2 months | Female | Right shoulder | D | N | No |
| 2 | 6 months | Female | Chin | D | N | No |
| 3 | 7 months | Female | Chest wall | D | N | No |
| 4 | 15 years | Male | Head,neck and chest wall | N | I | No |
| 5 | 3 years  10 months | Male | Chest wall | N | N | No |

Note: N, normal; I, increase; D, decrease.

Table 2. Imaging features of all patients

| Patients | US | | |  | CT | | | |
| --- | --- | --- | --- | --- | --- | --- | --- | --- |
|  | BLS | ATL | ST |  | LF | ST | FSS | PSISAT |
| 1 | Abundant | Yes | Yes |  | Diffuse | Yes | Yes | Yes |
| 2 | Abundant | Yes | Yes |  | Diffuse | Yes | Yes | Yes |
| 3 | Abundant | No | Yes |  | Focal | Yes | Yes | No |
| 4 | Patchy | No | Yes |  | Diffuse | Yes | Yes | Yes |
| 5 | Abundant | Yes | Yes |  | - | - | - | - |

Note: US, ultrasound; CT, computed tomography; BLS, blood flow signal; ATL, anechoic tubular lesion; ST,skin thickening; LF, lesion feature; FSS, flocculent soft-tissue shadow; PSISAT, punctate soft-tissue intensity within subcutaneous adipose tissue.
