# Supplementary material for: Clinical and imaging features of tufted angioma in children
Source: Front Pediatr. 2026 Jun 18;14:1756736. doi: 10.3389/fped.2026.1756736 (PMC13323123; doi:10.3389/fped.2026.1756736)
Supplement: Supplementary file 2 [file Table2.docx]

﻿For CT

The imaging protocol for CT were as follow: slice thickness, 5mm; tube voltage, 120kVp; tube current, adjusted automatically; matrix, 512 * 512; standard resolution algorithms. As for contrast-enhanced CT, images were acquired after injection of contrast agent. Contrast agent is Iohexol (50ml; 17.5g, GE Pharmaceuticals Shanghai Co., Ltd.), at a dose of 1.5-2.0 ml per kilogram of body weight.

For MRI

The imaging protocol for head or face (T2WI): TE, 100.00 ms; field of view, 16.0×16.0; acquisition matrix, 181×164; slice thickness, 3.0 mm; spacing between slices, 3.3 mm.

The imaging protocol for head or face (CET1): TE, 8.47 ms; field of view, 16.0×16.0; acquisition matrix, 181×164; slice thickness, 3.0 mm; spacing between slices, 3.3 mm.

The imaging protocol for neck (T2WI): TE, 84.70 ms; field of view, 19.0×19.0; acquisition matrix, 233×172; slice thickness, 3.0 mm; spacing between slices, 3.5 mm.

The imaging protocol for neck (CET1): TE, 8.79 ms; field of view, 19.0×19.0; acquisition matrix, 233×172; slice thickness, 3.0 mm; spacing between slices, 3.5 mm.

The contrast agent used in magnetic resonance imaging is gadotate dimeglumine (15 ml: 5.6535 g, Hainan Puli Pharmaceutical Co., Ltd.), at a dose of 0.2 ml per kilogram of body weight.
